# Short-term outcomes in robot-assisted compared to laparoscopic colon cancer resections: a systematic review and meta-analysis

**DOI:** 10.1007/s00464-021-08782-7

**Published:** 2021-11-01

**Authors:** Pedja Cuk, Mie Dilling Kjær, Christian Backer Mogensen, Michael Festersen Nielsen, Andreas Kristian Pedersen, Mark Bremholm Ellebæk

**Affiliations:** 1 Surgical Department, University Hospital of Southern Jutland, Kresten Philipsens Vej 15, 6200 Aabenraa, Denmark; 2 Research Unit for Surgery, Odense University Hospital and University of Southern Denmark, Odense, Denmark; 3 Institute of Regional Health Research, University of Southern Denmark, Odense, Denmark

**Keywords:** Robot-assisted surgery, Colon cancer, Laparoscopy, Minimally invasive surgery

## Abstract

**Background:**

Robot-assisted surgery is increasingly adopted in colorectal surgery. However, evidence for the implementation of robot-assisted surgery for colon cancer is sparse. This study aims to evaluate the short-term outcomes of robot-assisted colon surgery (RCS) for cancer compared to laparoscopic colon surgery (LCS).

**Methods:**

Embase, MEDLINE, and Cochrane Library were searched between January 1, 2005 and October 2, 2020. Randomized clinical trials and observational studies were included. Non-original literature was excluded. Primary endpoints were anastomotic leakage rate, conversion to open surgery, operative time, and length of hospital stay. Secondary endpoints were surgical efficacy and postoperative morbidity. We evaluated risk of bias using RoB2 and ROBINS-I quality assessment tools. We performed a pooled analysis of primary and secondary endpoints. Heterogeneity was assessed by *I*^2^, and possible causes were explored by sensitivity- and meta-regression analyses. Publication bias was evaluated by Funnel plots and Eggers linear regression test. The level of evidence was assessed by GRADE.

**Results:**

Twenty studies enrolling 13,799 patients (RCS 1740 (12.6%) and LCS 12,059 (87.4%) were included in the meta-analysis that demonstrated RCS was superior regarding: anastomotic leakage (odds ratio (OR) = 0.54, 95% CI [0.32, 0.94]), conversion (OR = 0.31, 95% CI [0.23, 0.41]), overall complication rate (OR = 0.85, 95% CI [0.73, 1.00]) and time to regular diet (MD =  − 0.29, 95% CI [− 0.56, 0.02]). LCS proved to have a shortened operative time compared to RCS (MD = 42.99, 95% CI [28.37, 57.60]). Level of evidence was very low according to GRADE.

**Conclusion:**

RCS showed advantages in colonic cancer surgery regarding surgical efficacy and morbidity compared to LCS despite a predominant inclusion of non-RCT with serious risk of bias assessment and a very low level of evidence.

**Supplementary Information:**

The online version contains supplementary material available at 10.1007/s00464-021-08782-7.

Minimally invasive technology has undergone a rapid advancement within the last 20 years introducing robot-assisted colon surgery (RCS). First reports of RCS were described by Weber et al. [1]. The method was implemented in order to simplify dissection in the narrow pelvis and improve ergonomics for the surgeon. Due to a technological progress within the last decade, robot-assisted surgery has minimized tremor and enhanced camera guidance, which may have improved and simplified the dissection [2–5]. Laparoscopic colon surgery (LCS) is associated with a reduced overall morbidity, shortened hospital stay, minimized analgesics consumption, faster recovery of bowel function and oral intake compared to open surgery [6–8]. The method results in less manipulation of the intra-abdominal organs and contributes to a lower surgical stress response [9–11]. To our knowledge benefits of RCS compared to LCS have not been examined in randomized controlled trials with the exception of Park et al. [12]. No difference in intra- or postoperative outcomes was demonstrated when the RCS was compared to LCS in a study based on a small number of patients (*n* = 70) [12]. However, a prolonged operation time and higher procedure costs were observed in RCS.

Present literature that compares RCS and LCS includes a mixed population of benign and malignant colorectal surgery [13–15]. Systematic reviews and meta-analyses have shown a favorable outcome for the intracorporeal anastomosis technique when assessed for intra- versus extracorporeal anastomosis technique in colectomies for right-sided colon cancer [16]. Besides lower morbidity rates in right-sided colectomies performed by RCS versus LCS in a heterogenous population, to our knowledge, no meta-analyses has been conducted to determine overall effects of RCS in colonic cancer surgery [15, 16]. It would be relevant to perform a systematic review and, if possible, a meta-analysis on the effectiveness and safety of RCS versus LCS in a patient cohort with only colon cancer. In addition to the main analysis, a supplemental sensitivity- and meta-regression analysis is likely to be conducted to demonstrate any potential subgroup-influence on the overall analysis.

We aim to evaluate the short-term outcomes of robot-assisted colon resection for cancer compared to conventional laparoscopy in this systematic review and meta-analysis. Primary endpoints are anastomotic leakage rate, conversion to open surgery, operative time and length of hospital stay.

## Methods

The study was performed in accordance with the Preferred Reporting Items for Systematic Reviews and Meta-Analyses (PRISMA) guidelines and Meta-analysis of Observational Studies in Epidemiology (MOOSE) checklist [17, 18] (Supplemental Digital Content Material 1, Tables 1, 2). The first author registered the study protocol in PROSPERO, University of York (CRD42020211681).

### Search strategy

**Fig. 1:**
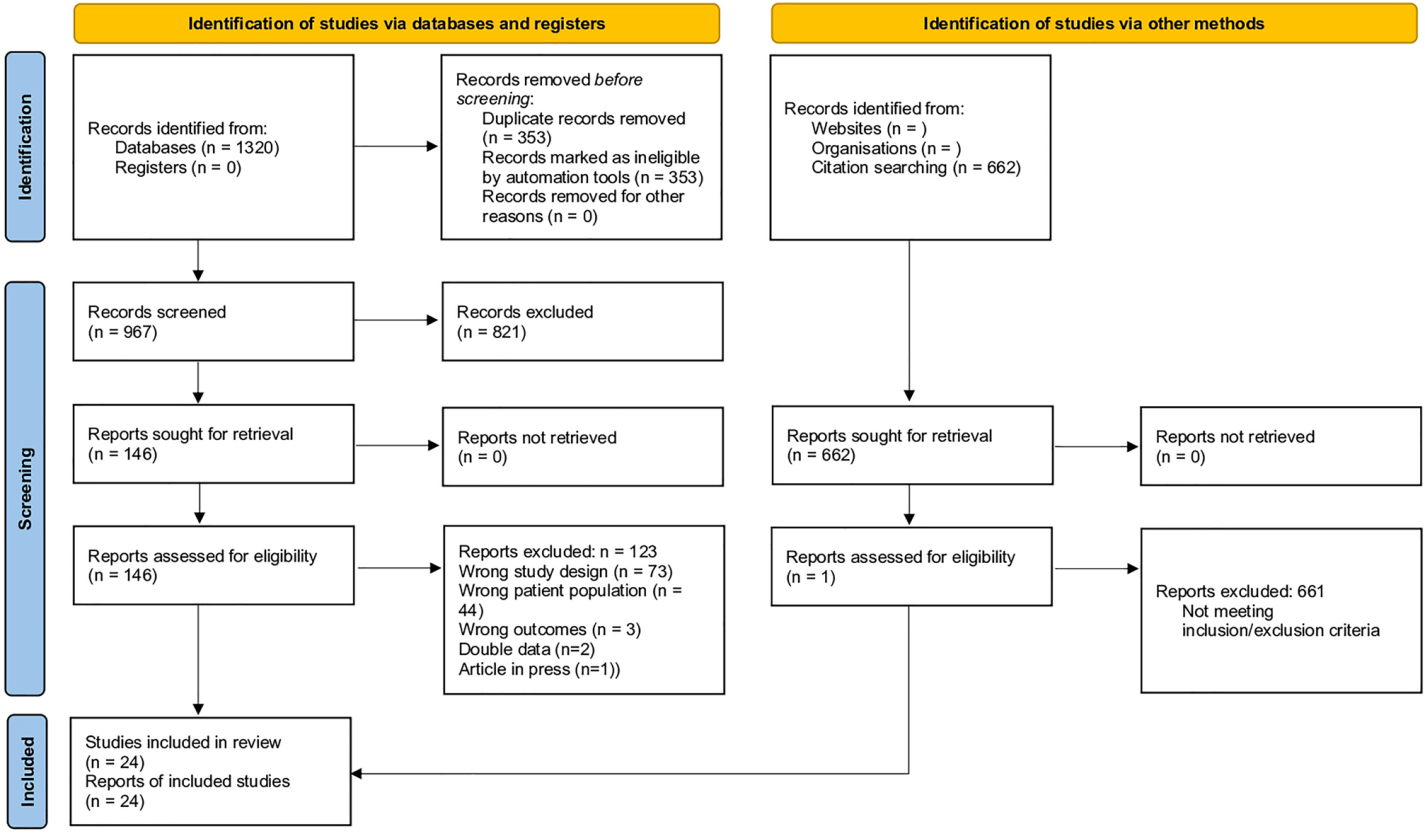
PRISMA flowchart of study selection process

A systematic literature search was performed with assistance of a medical research librarian, University of Southern Denmark in databases: Embase, MEDLINE, and Cochrane Library (CENTRAL). Following terms were included in the search strategy: “colonic neoplasms”, “colon cancer”, “colon tumor”, “colon carcinoma”, “laparoscopy” and “robot-assisted surgery”. The exact literature search strategy included articles published between January 1, 2005 and October 2, 2020, provided in Supplemental Digital Content Material 1, Fig. 1. The time frame was selected as the first case series of RCS were published in 2002 [1], and we expected high-volume comparative studies on LCS and RCS to be relatively sparse within the first four years due to gradual implementation. Two independent authors (PC and MD) screened and selected all articles. In case of disagreement, a third independent senior author (MBE) was available for consultation. The first author imported the literature from databases into the review program Covidence®, *Covidence systematic review software, Veritas Health Innovation, Melbourne, Australia*. A manual search for additional relevant articles was obtained from the reference list of included studies. Literature not available in the searched databases and by article review was obtained through direct correspondence to the main authors.

### Eligibility criteria

Inclusion criteria were (1) comparative literature for elective colon cancer resections performed robot-assisted and laparoscopically, (2) colorectal studies if results were reported independently for colon surgery, and (3) further, we arbitrarily decided that studies must include a minimum 15 patients in both groups. Only studies published in English were included. Included study designs were randomized clinical trials (RCT) and observational studies. A preliminary search had indicated only few RCTs existed of that time, which to a modest degree would increase the level of evidence in a comprehensive systematic review and meta-analysis. The following studies were excluded in case of: duplicates, abstracts, cover letters, editorials, reviews, commentaries, case reports and pediatric studies. Studies based on a cohort of palliative colorectal resections, combined benign and malignant colorectal surgery not reporting the results independently or in case of emergent surgery were also excluded.

### Outcomes and data extraction

Primary outcomes were anastomotic leakage rate, conversion from robot-assisted or laparoscopic to open surgery, operative time and length of hospital stay. Secondary outcomes included overall complication rates, time to first flatus and oral diet, intraoperative blood loss, rate of postoperative bleeding, surgical site infection, intra-abdominal abscess formation, postoperative bowel obstruction, the total amount of harvested lymph nodes, 30 days mortality, Clavien–Dindo classification I–V, surgical and medical complications [19]. Data were extracted by PC into a priori developed data extraction form. Following extraction were performed regarding study data: authors, year of publication, study design, country of study, study period, risk of bias and quality of evidence assessments. We extracted following demographic data: sex, age, BMI, ASA-score ≥ 3, T-stage ≥ 3, location of tumor, type of surgical procedure and if previous abdominal surgery was performed. Data referring to predefined primary and secondary endpoints were also extracted.

### Risk of bias assessment and quality of evidence (GRADE)

Three reviewers (PC, MD and AKP) performed the risk of bias assessment by using the Cochrane handbook risk of bias (RoB2—2019) and (ROBINS-I tool—2016) [20, 21]. The first author used an electronic program, *Robvis*, to create quality assessment and weighted bar plots to provide better visualization of the individual studies [22]. PC and AKP assessed the certainty of evidence by applying “The Grading of Recommendations Assessment, Development and Evaluation (GRADE)” approach [23]. A summary of findings table was performed on the most important clinical outcomes with the assistance of GRADEpro GDT software (*GRADEpro GDT: GRADEpro Guideline Development Tool [software]. McMaster University, 2020 (developed by Evidence Prime, Inc.). Available from *https://gradepro.org*)*.

### Statistical analysis

For binary variables, odds ratios (OR) and corresponding 95% confidence interval were calculated for each study. Continuous variables reported in median and range, were converted to mean and standard deviation as suggested by Hozo et al. [24]. Subsequently, mean differences and their corresponding 95% confidence intervals were calculated for each study. We used Higgins’ I square statistics (*I*^2^) in order to examine the variation in heterogeneity across studies [25]. Values < 30% were associated with low heterogeneity, > 50% with substantial heterogeneity, while studies > 75% *I*^2^ were considerable heterogenic, as described in the Cochrane handbook for systematic reviews of interventions [26]. We used a fixed-effect model (Mantel–Haenszel method) in studies associated with low heterogeneity (< 50%). Random-effect models were utilized according to Der Simonian–Laird method in studies with high heterogeneity (> 50%). A *p*-value < 0.05 was considered as statistically significant. We performed subgroup analyses and computed results from right-sided colectomies separately. In order to explain a possible implication of substantial and considerable heterogeneity (> 50%) found in our primary and secondary outcomes from the main analysis, we conducted a sensitivity analysis on right-sided resections. By visual inspection of forest and funnel plots, we identified and excluded outlier studies with extreme effect sizes. A random-effects meta-regression analysis was performed to examine a possible moderator effect on primary outcomes. We used baseline study and clinical moderators (year of study publication, age, male sex, BMI, T-stage ≥ 3, anastomosis techniques (intra- or extracorporeal), and history of previous abdominal surgery). Publication bias were assessed by visual examination for any possible asymmetry presented in funnel plots and Egger’s linear regression test [27, 28]. A significance level of (*p* < 0.05) was correlated to asymmetry and signs of publication bias. All statistical analysis were performed with STATA—*StataCorp. 2019. Stata Statistical Software: Release 16. College Station, TX: StataCorp LLC* in cooperation with a biostatistician (AKP).

## Results

### Systematic review

The systematic literature search yielded 1321 articles. After removal of duplicates, 966 articles were screened for title and abstract. Of these, 146 articles were included for full text screening, and subsequently 20 articles were included in the quantitative analysis [12, 29–31, 33, 35, 36, 38, 39, 41–51]. The complete search strategy is presented according to PRISMA guidelines in Fig. 1.

### Study characteristics

A total of 1862 patients (13%) underwent RCS and 12,231 (87%) LCS. The majority of the included studies were of observational design [29–51] in addition to one RCT study by Park et al. [12]. Table 1 shows an overview of the included studies.

**Table 1:** Study characteristics

Author country Y.O. publ. study period	N.O. patients RCS/LCS	Anastomosis technique, RCS/LCS (EC/IC/NA), *n* (%)	Age (years), mean ± SD, RCS/LCS	BMI, mean (kg/m^2^) RCS/LCS	Study type	Tumor location
Bertani, Italy, 2011, 2009–2010 [29]	34/30	IC and EC	62.5 ± 8.4/62.0 ± 10.2	26.1 ± 3.71/24.6 ± 3.54	Prospective	Right and left colon
Park, Korea, 2012, 2009–2011 [12]	35/35	RCS − EC: 5 (14), RCS − IC: 30 (86), LCS − EC: 28 (80), LCS − IC: 7 (20)	62.8 ± 10.5/66.5 ± 11.4	24.4 ± 2.5/23.8 ± 2.7	RCT	Right colon
Lim, Korea, 2012, 2006–2008 [30]	34/146	Only EC	59.6 ± 8.4/59.7 ± 11.5	24.8 ± 2.1/23.8 ± 3.8	Retrospective	Left colon
Helvind, Denmark, 2013, 2009–2012 [31]	101/162	Only EC	72.2 (39–93)/75.3 (47–96)	24.6 (16.8–36.0)/24.9 (15.2–45.7)	Retrospective	Right and left colon
Morpurgo, Italy, 2013, 2008–2012 [32]	48/48	RCS − IC: 48 (50), LCS − EC: 48 (50)	68.8 ± 8/74 ± 11	25 ± 3.5/28 ± 4	Retrospective	Right colon
de'Angelis, France, 2015, 2010–2014 [33]	22/22	Only EC	72.18 ± 10.79/71 ± 10.14	24.12 ± 2.64/24.28 ± 2.7	Prospective	Transverse colon
Guerrieri, Italy, 2015, 2013–2014 [34]	24/23	RCS − EC: 5 (21), RCS − IC: 19 (79), LCS − EC: 11 (48), LCS − IC: 12 (52)	69.5 (57–80)/65.5 (57–75)	26 (23–28)/26.5 (23–29)	Retrospective	Right and left colon
Kang, Korea, 2016, 2007–2011 [35]	20/43	Only EC	66.0 ± 9.6/65.7 ± 13.2	23.5 ± 2.4/23.0 ± 3.0	Retrospective	Right colon
Jung, Korea, 2016, 2007–2013 [36]	51/161	NR	60.2 ± 9.5/60.3 ± 11.3	23.5 ± 3.0/23.2 ± 2.8	Retrospective	Right and left colon
de'Angelis, France, 2016, 2012–2015 [37]	30/50	Only EC	71 ± 8.5/71.1 ± 12.92	26.43 ± 3.21/25.26 ± 4.19	Prospective	Right colon
Cardinali, Italy, 2016, 2013–2015 [38]	30/60	RCS − IC: 30 (33), LCS − EC: 60 (67)	68.67 ± 12.93/70.78 ± 9.56	25.44 ± 4.30/26.36 ± 3.17	Retrospective	Right colon
Widmar, USA, 2017, 2012–2014 [39]	119/163	NR	68 (58–77)/64 (54–75)	28 (24–32)/29 (25–32)	Retrospective	Right colon
Kim, Korea, 2018, 2012–2017 [40]	20/51	Only EC	58 ± 10/56 ± 13	25.5 ± 3.8/24 ± 3.0	Retrospective	Left colon
Scotton, Italy, 2018, 1998–2017 [41]	206/160	RCS − IC: 206 (56), LCS − EC: 160 (44)	70.1 ± 11.7/70.3 ± 12.7	26.0 ± 3.7/25.6 ± 5.9	Retrospective	Right colon
Ngu, Singapore, 2018, 2015–2017 [42]	16/16	Only IC	68.6 ± 10.9/69.6 ± 9.6	23.7 ± 3.8/24.7 ± 4.2	Retrospective	Right colon
Spinoglio, Italy, 2018, 2005–2013 [43]	100/100	Only IC	71.2 ± 10.2/71.2 ± 10.6	25.1 ± 4/25.8 ± 4.4	Retrospective	Right colon
Mégevand, Italy, 2018, 2010–2015 [44]	50/50	RCS − IC: 49 (98), RCS − EC: 1 (2), LCS − IC: 18 (36), LCS − EC: 32 (64)	70.3/69.6	26.2/25.25	Retrospective	Right colon
Fransgaard, Denmark, 2018, 2010–2015 [45]	511/8104	NR	NE	NE	Retrospective	Right and left colon/rectum
Haskins, USA, 2018, 2012–2014 [46]	89/2405	NR	68.9 ± 11.8/68.3 ± 12.2	29.3 ± 6.3/28.5 ± 6.3	Retrospective	Right colon
Polat, Netherlands, 2019, 2014–2017 [47]	129/138	RCS − IC: 129 (48), LCS − IC + EC: 138 (52) depending on the surgeon's choice	NE	NE	Retrospective	Right and left colon/rectum
Merola, Italy, 2019, 2012–2017 [51]	94/94	RCS − IC: 94 (50), LCS − IC: 94 (50)	69.41 ± 10.31/72.09 ± 9.54	26.94 ± 4.91/27.97 ± 5.73	Retrospective	Right colon
Yozgatli, Turkey, 2019, 2015–2017 [48]	35/61	RCS− IC: 35 (36), LCS − IC and EC 61 (54) based on surgeon's choice	65 ± 13/65 ± 13	29 ± 5/27 ± 5	Retrospective	Right colon
Ozben, Turkey, 2020, 2011–2018 [49]	38/80	RCS − IC: 33 (86.8), RCS − EC: 4 (10.5), RCS − NA: 1 (2.6), LCS − IC: 16 (20.0), LCS − EC: 60 (75), LCS − NA: 4 (5.0)	62.3 ± 15.7/64.1 ± 15.5	25.3 ± 4.5/26.7 ± 5.7	Retrospective	Transverse colon
Ceccarelli, Italy, 2020, 2014–2019 [50]	26/29	Only IC	69.1 ± 9.4/75.0 ± 11.7	24.4 ± 3.8/24.2 ± 2.8	Retrospective	Right colon
Total N.O. patients	1862 (13%)/12,231 (87%)					

*Y.O*. publ year of publication, *N.O*. number of, *RCS* robot-assisted colon surgery, *LCS* laparoscopic colon surgery, *EC* extracorporeal, *IC* intracorporeal, *NA* no anastomosis, *RCT* randomized controlled trial, *SD* standard deviation, *NR* not reported, *NE* not estimable

### Risk of bias assessment

The RCT study by Park had a low risk of bias regarding randomization, missing outcome data and the selection of reported results. There was a moderate risk of bias related to deviations from intended interventions and measurements of the outcome. Due to the heterogeneous cohort of included patients from the observational studies, there was a predominantly serious risk of bias regarding confounding and selection of participants, as presented in Supplemental Digital Content Material 2, Fig. 1.

### Meta-analysis

#### Patient demographic

We included 20 out of 24 studies from the systematic literature search in the meta-analysis [12, 29–31, 33, 35, 36, 38, 39, 41–51] with a total of 13,799 patients [1740 (12.6%) underwent RCS and 12,059 (87.4%) LCS]. Significant differences in preoperative patient characteristics were determined between the RCS and LCS group regarding previous abdominal surgery [12, 29–31, 33, 35, 38, 41, 43, 44, 48, 49, 51] and male gender [12, 29–31, 33, 35, 36,38, 39, 41–46, 48–51]. Table 2 summarizes patient demographics. There was no statistically significant difference in preoperative characteristics between the two groups regarding age, BMI, tumor stage (T3 ≥ 3) or ASA-score ≥ 3.

**Table 2:** Meta-analysis of patient characteristics in the included studies

Outcome	N.O. Studies	RCS	LCS	OR/MD 95% CI	*I*^2^ (%)	*p*-value
Age (SD)	17	66.87 (10.31)	67.69 (10.81)	− 0.67 [− 2.46, 1.11]	80.85	0.46
BMI (SD)	17	25.54 (3.58)	25.49 (4.03)	0.02 [− 0.47, 0.51]	63.54	0.95
Male gender (%)	19	918 (56.9)	5960 (50.0)	1.31 [1.16, 1.47]	59.07	0.00
Previous abdominal surgery (%)	13	246 (30.8)	344 (32.9)	0.76 [0.62, 0.95]	0.00	0.01
ASA ≥ 3 (%)	16	286 (30.2)	1629 (45.3)	0.94 [0.77, 1.14]	26.60	0.53
T-stage ≥ 3 (%)	18	831 (54.9)	6736 (71.7)	0.90 [0.79, 1.04]	0.00	0.14

*N.O*. number of, *RCS* robot-assisted colon surgery, *LCS* laparoscopic colon surgery, *OR* odds ratio, *MD* mean difference, *CI* = confidence interval, *I*^2^ heterogeneity

#### Primary outcome: anastomotic leakage

**Fig. 2:**
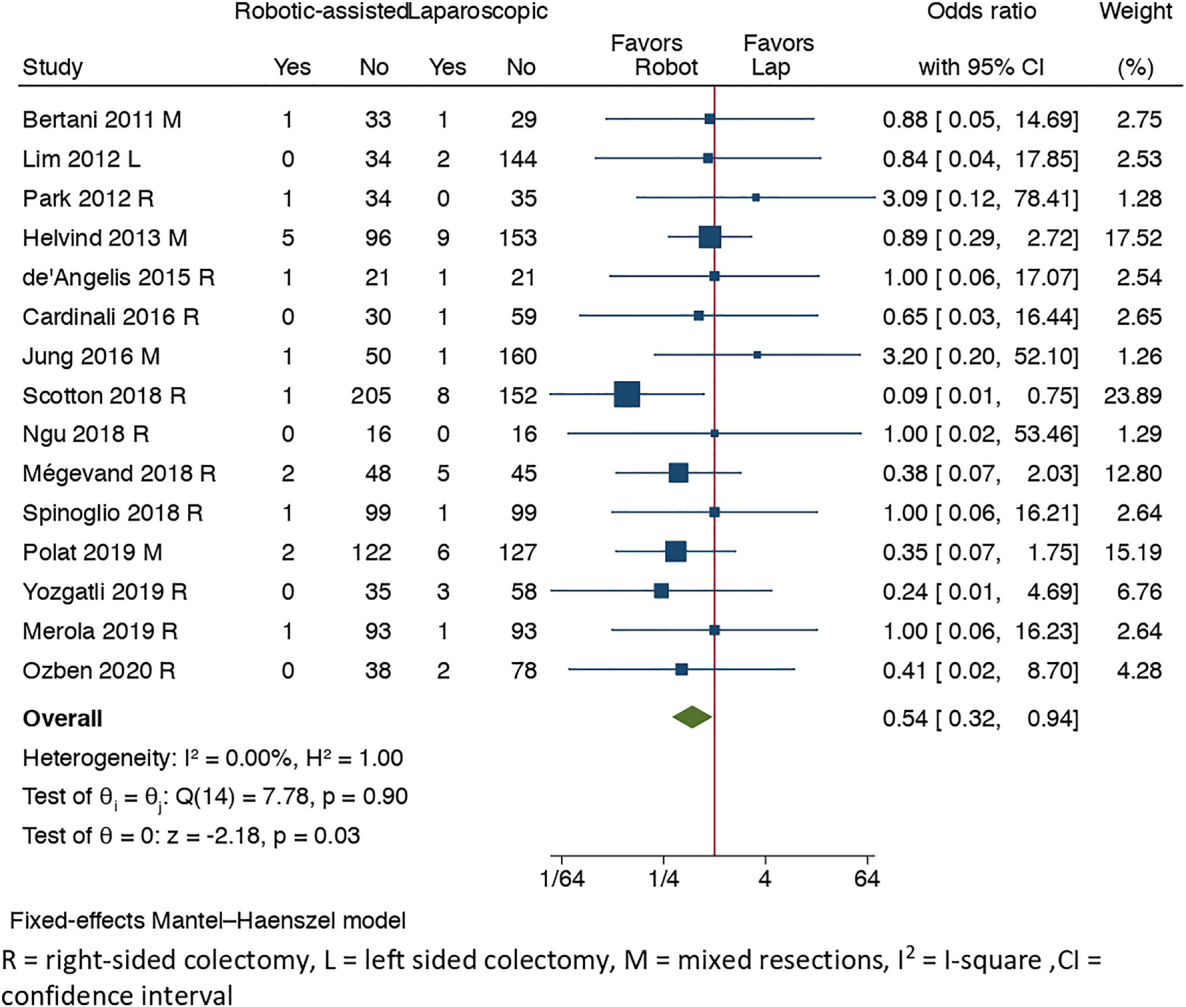
Forest plot of anastomotic leakage

Anastomotic leakage rate was reported in 15 studies [12, 29–31, 33, 36, 38, 41–44, 47–49, 51], with 16 events out of 970 (1.65%) in the RCS group and 41 out of 1310 (3.13%) in LCS group with a significant difference in favor of RCS (OR = 0.54, 95% CI [0.32, 0.94], *I*^2^ = 0%, *p* = 0.03). There was a tendency for a reduction in anastomotic leakage rate in the RCS group especially after year 2018 [41, 44, 47–49] according to Fig. 2.

#### Primary outcome: conversion to open surgery

**Fig. 3:**
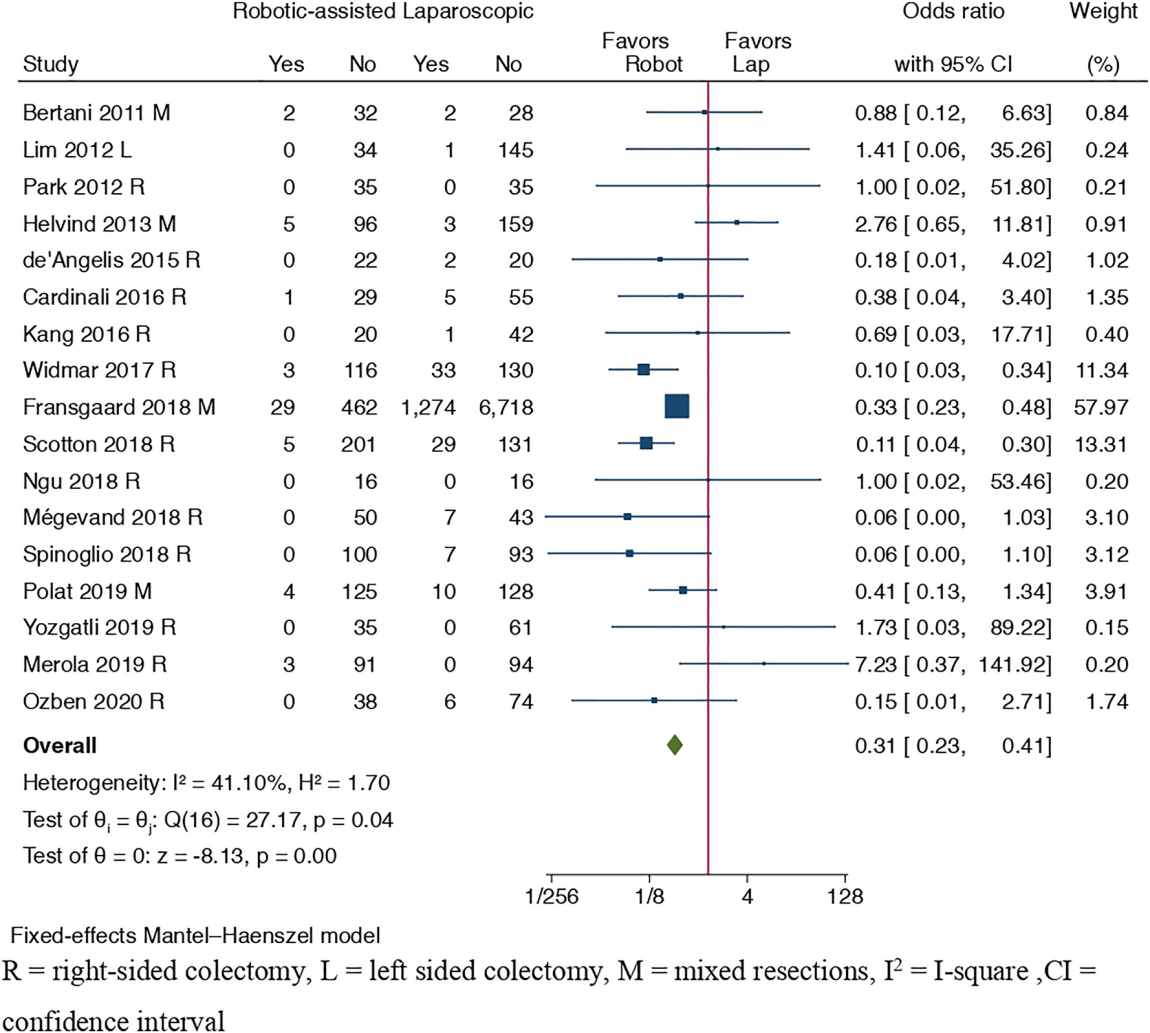
Forest plot of conversion rate to open surgery

Conversion rates were reported in 17 studies [12, 29–31, 33, 35, 38, 39, 41–45, 47–49, 51]. There was an advantage for lower conversion rates in RCS versus LCS group (OR = 0.31, 95% CI [0.23, 0.41], *I*^2^ = 41.10%, p = 0.00), see Fig. 3.

#### Primary outcome: operative time

Operative time was prolonged in RCS compared to LCS group (MD = 42.99, 95% CI [28.37, 57.60], *I*^2^ = 96.99%, *p* = 0.00). During the inclusion period the operative time for RCS was unchanged and showed no decreasing tendency among included studies (*n* = 19) [12, 29–31, 33, 35, 36, 38, 39, 41–44, 46–51]. Forest plot of operative time is presented in Supplemental Digital Content Material 3, Fig. 1.

#### Primary outcome: length of hospital stay

**Fig. 4:**
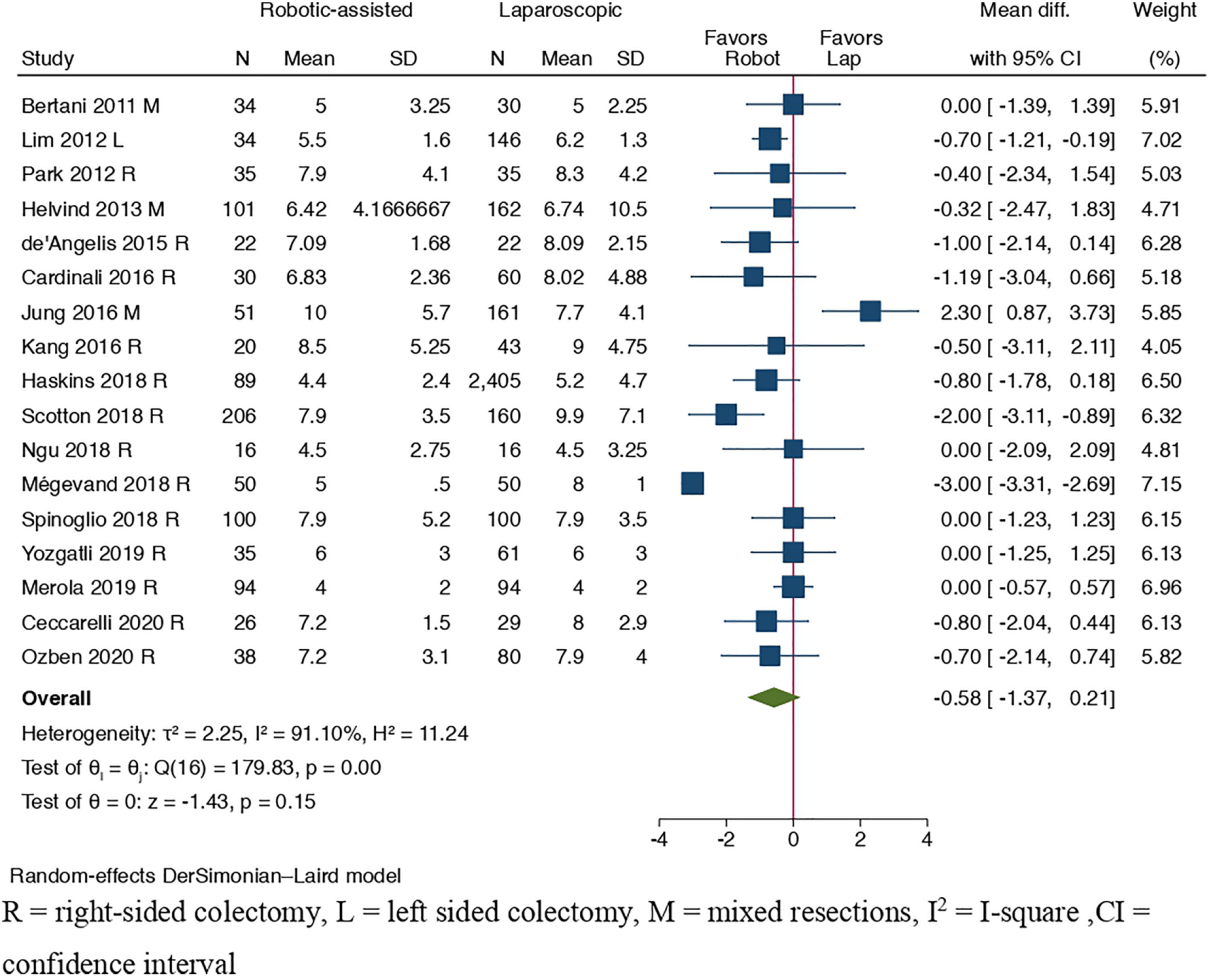
Forest plot of length of hospital stay

Length of hospital stay was reported in 17 studies [12, 29–31, 33, 35, 36, 38, 41–44, 46, 48–51] presented in Fig. 4, and did not differ significantly between the two surgical methods (MD =  − 0.58, 95% CI [− 1.37, 0.21], *I*^2^ = 91.10%, *p* = 0.15).

#### Secondary outcomes

Data from overall complication rates were reported in 20 studies [12, 29–31, 33, 35, 36, 38, 39,41–51] in which 303 complications out of 1740 (17.41%) occurred in the RCS group and 2051 out of 12,059 (17.01%) in LCS group. There was a significant lower difference in the pooled OR regarding the overall complication rate in favor of RCS (OR = 0.85, 95% CI [0.73, 1.00], *I*^2^ = 9.75%, *p* = 0.04). A total of 11 studies [12, 29, 30, 33, 35, 38, 41–43, 49, 50] reported a statistically significant difference in time to regular diet in favor of RCS (MD =  − 0.29, 95% CI [− 0.56, − 0.02], *I*^2^ = 58.17%, *p* = 0.04). We detected no differences regarding following outcomes: time to first flatus, intra-abdominal abscess rate, Clavien–Dindo complication rate I–III or IV–V, medical complications, postoperative bleeding, ileus, wound abscess, amount of lymph node harvest, estimated intraoperative blood loss or 30 days mortality rate (see Supplemental Digital Content Material 4, Figs. 1, 2, 3, 4, 5, 6, 7, 8, 9, 10, 11, 12, 13).

### Subgroup analysis: right-sided hemicolectomies

Fourteen studies [12, 33, 35, 38, 39, 41–44, 46, 48–51] with a total number of 4198 patients (RCS = 880 (20.97%), LCS = 3318 (79.01%) underwent right-sided hemicolectomy. Conversion rates were lower in the RCS compared to the LCS group (OR = 0.18, 95% CI = [0.10, 0.30], *I*^2^ = 12.72%, *p* = 0.00). The rate of anastomotic leakage was reduced in the RCS group (OR = 0.41, 95% CI [0.19, 0.88], *I*^2^ = 0%, *p* = 0.02). The amount of overall complication rates did not differ significantly between RCS and LCS group (OR = 0.85, 95% CI [0.69, 1.04], *I*^2^ = 25.26%, *p* = 0.11). Mean difference in operative time favorized LCS with considerable and significant heterogeneity (MD = 57.45, 95% CI [38.47, 76.42], *I*^2^ = 94.46%, *p* = 0.00). A significant reduction of length of stay with substantial heterogeneity was demonstrated in the RCS group (MD =  − 0.91, 95% CI [− 1.60, − 0.22], *I*^2^ = 73.74%, *p* = 0.01). No difference between the surgical methods was found regarding to following outcomes: medical complications, Clavien–Dindo Complication rate I–V, 30 days mortality rate, abdominal abscess, wound abscess, postoperative bleeding, postoperative ileus, intraoperative blood loss, amount of harvested lymph nodes, time to regular diet or time to first flatus, presented in Supplemental Digital Content Material 5, Table 1.

### Sensitivity analysis

We performed a supplementary sensitivity analysis on outcomes that initially presented with substantial or considerable heterogeneity (*I*^2^ > 50%) from main analyses. A significant advantage was found for RCS compared to LCS regarding length of stay (MD =  − 0.45, 95% CI [− 0.73, − 0.17], *I*^2^ = 0%, *p* = 0.00), amount of harvested lymph nodes (MD =  − 0.96, 95% CI [− 1.79, − 0.14], *I*^2^ = 0%, *p* = 0.02) and intraoperative blood loss (MD =  − 17.14, 95% CI [− 24.41, − 9.87], *I*^2^ = 19.10%, *p* = 0.00). Operative time was in favor of LCS (MD = 41.99, 95% CI [35.01, 48.96], *I*^2^ = 0%, *p* = 0.00). Time to regular diet and time to first flatus did not differ between the two surgical methods. See Supplemental Digital Content Material 6, Table 1.

### Sensitivity analysis of studies with low risk of bias

We included studies with low risk of bias and performed a sensitivity analysis across all outcomes. Conversion rates were in favor of RCS compared to LCS (OR = 0.31, 95% CI [0.22, 0.43], *I*^2^ = 50.17%, *p* = 0.00). Except for shortened operative time in favor of LCS (MD = 71.00, 95% CI [16.81, 125.19], *I*^2^ = 98.56%, *p* = 0.01), no differences were observed across other outcomes between the RCS and LCS. See Supplemental Digital Content Material 7, Table 1.

### Meta-regression analysis

Meta-regression analysis was performed in order to clarify a potential moderator effect on primary outcomes. We found following variables had a modifying effect on conversion rates in favor of robot-assisted surgery: increasing age (*β* =  − 0.407, 95% CI [− 0.727, − 0.087], *p* = 0.013), and a more recent year of publication (*β* =  − 0.225, 95% CI [− 0.452, 0.002], *p* = 0.052. Male gender (*β* = 0.431, 95% CI [0.045, 0.818], *p* = 0.029) was an independent factor and favored conversion rates in the LCS group. The intracorporeal anastomosis technique was associated with a faster operative time in the RCS compared to the LCS group (*β* =  − 0.860, 95% CI [− 1.576, − 0.144], *p* = 0.019), while a more recent year of publication favored operative times in the LCS group (*β* = 0.154, 95% CI [− 0.003, 0.312], *p* = 0.054). The remaining co-variates did not have a moderating effect on other primary outcomes (anastomotic leakage or length of stay), see Supplemental Digital Content Material 8, Table 1.

### GRADE (quality of evidence) and publication bias assessment

The quality of evidence was applied by GRADE assessment in the most important clinical outcomes (anastomotic leak, overall complication rate, conversion rate, intraoperative blood loss, length of hospital stay, Clavien–Dindo complication grade IV–V and 30 days mortality rate). We found the quality of evidence very low across all outcomes of interest mainly due to a serious risk of bias and/or inconsistency, see Table 3. In addition to the amount of intraoperative blood loss and harvested lymph nodes the included studies showed no signs of publication bias assessed by Funnel plots and Egger’s regression test presented in Supplemental Digital Content Material 9, Table 1 and Figs. 1, 2, 3, 4, 5, 6, 7, 8, 9, 10, 11, 12, 13, 14, 15, 16 and 17.

**Table 3:** GRADE quality of evidence assessment of most important clinical outcomes in robot-assisted to laparoscopic colon cancer surgery

Participants (studies)											
	Risk of bias	Inconsistency	Indirectness	Imprecision	Publication bias	Overall certainty of evidence	Study event rates (%)		Relative effect (95% CI)	Anticipated absolute effects	
							With laparoscopic surgery	With robot-assisted		Risk with laparoscopic surgery	Risk difference with robot-assisted
Anastomotic leak											
2280 (15 Observational studies)	Serious^a^	Not serious	Not serious	Serious^b^	None	⨁◯◯◯ VERY LOW	41/1310 (3.1%)	16/970 (1.6%)	OR 0.54 (0.32 to 0.94)	31 Per 1000	14 Fewer per 1000 (from 21 to 2 fewer)
Overall complication rate											
13,799 (20 Observational studies)	Very serious^c^	Not serious	Not serious	Not serious	None	⨁◯◯◯ VERY LOW	2051/12,059 (17.0%)	303/1740 (17.4%)	OR 0.85 (0.73 to 1.00)	170 Per 1000	22 Fewer per 1000 (from 40 to 0 fewer)
Conversion											
10,906 (17 Observational studies)	Serious^a^	Not serious	Not serious	Not serious	None	⨁◯◯◯ VERY LOW	1380/9352 (14.8%)	52/1554 (3.3%)	OR 0.31 (0.23 to 0.41)	148 Per 1000	97 Fewer per 1000 (from 109 to 81 fewer)
Intraoperative blood loss											
635 (8 Observational studies)	Serious^a^	Serious^d^	Not serious	Not serious	Publication bias strongly suspected ^e^	⨁◯◯◯ VERY LOW	417	218	–	The mean intraoperative blood loss was 83.8 mL	MD 0.33 lower (16.54 lower to 15.88 higher)
Length of hospital stay											
4635 (17 Observational studies)	Very serious^c^	Very serious^f^	Not serious	Not serious	None	⨁◯◯◯ VERY LOW	3654	981	-	The mean length of hospital stay was 7.09 days	MD 0.58 lower (1.37 lower to 0.21 higher)
Clavien–Dindo grade IV–V											
1041 (8 Observational studies)	Serious^a^	Not serious	Not serious	Very serious^b^	None	⨁◯◯◯ VERY LOW	27/578 (4.7%)	15/463 (3.2%)	OR 0.73 (0.38 to 1.37)	47 Per 1000	12 Fewer per 1000 (from 28 fewer to 16 more)
Mortality											
4754 (15 Observational studies)	Serious^a^	Not serious	Not serious	Very serious^b^	None	⨁◯◯◯ VERY LOW	18/3657 (0.5%)	3/1097 (0.3%)	OR 0.76 (0.35 to 1.67)	5 Per 1000	1 Fewer per 1000 (from 3 fewer to 3 more)

*CI* confidence interval, *OR* odds ratio, *MD* mean difference

^a^Risk of bias assessment on outcome level downgraded by one level due to serious limitations

^b^Total number of events < 400 (small optimal information size)

^c^Risk of bias assessment on outcome level downgraded by two levels due to very serious limitations

^d^Substantial heterogeneity

^e^Funnel plot suggest heterogeneity

^f^Considerable heterogeneity

## Discussion

To our knowledge, this systematic review and meta-analysis is the first conducted study to analyze robot-assisted compared to laparoscopic surgery for elective colonic cancer resections. It included 14,093 patients and aimed to evaluate the short-term clinical outcomes by the two surgical methods. Our study indicated that RCS was superior to LCS in terms of anastomotic leakage rate, overall complication rate, conversion to open surgery and time to regular diet. Despite a lack of prospective studies in the meta-analysis, our main results were affirmed by supplementary statistical analyses. These indicated robot-assisted method had several clinical benefits compared to laparoscopy in predominantly right-sided tumors, which may defend its standardized implementation for the future treatment of right-sided colonic cancer.

The development of minimally invasive surgery caused an increased transition from LCS to RCS in the last decade. However, our review found only one RCT on this topic, and the non-randomized studies included were of very low quality of evidence. Therefore, no high-quality scientific evidence for the routine use of RCS exists. Nevertheless, our results consistently favorized RCS in relation to several primary and secondary outcomes.

A reduction in anastomotic leakage rate may have multiple causes. Surgery performed in our study included two different anastomosis techniques (extra- or intracorporeal). In a supplementary meta-regression analysis the anastomotic technique did not have a modifying effect on the anastomotic leakage rate. Previous systematic literature and meta-analyses compared the anastomotic leakage rate in intra- and extracorporeal anastomoses techniques in minimally invasive colon surgery with divergent conclusions [16, 52–54]. Possible causes of an increased leakage rate in extracorporeal anastomosis techniques are caused by a greater mobilization and traction on the bowel mesentery [53]. This manipulation may induce a decreased blood supply to the anastomosis, with an increased risk of leakage. There is also a risk of injury to bowel serosa, and increased risk of perforation in the extracorporeal anastomosis technique [16, 53]. A recent study by Emile et al. compared intra- and extracorporeal anastomoses techniques for benign and malignant conditions in minimally invasive right-sided colectomies. The rate of anastomotic leakage favorized the intracorporeal technique [16].

The risk of conversion in RCS versus LCS for colonic cancer surgery has been debated in previous meta-analyses with a general trend of lower conversion rates in RCS for right-sided resections [14, 15, 55, 56]. No significant impact on conversion rates in the RCS group was demonstrated in the meta-regression analysis despite a statistical overrepresentation of patients who previously had undergone abdominal surgery. Besides the ROLARR study [57], the risk of conversion to open surgery from RCS or LCS has not been investigated in a prospective, randomized design. The ROLARR study was conducted on rectal cancer resections and found no differences in conversion rates between groups. However, the study reported a significant difference in male patients with higher BMI in favor of RCS. In a recent meta-analysis by Wee et al. there was no divergence in the risk of conversion in obese patients between the two surgical methods [58]. No preoperative patient selection in either of the surgical methods occurred in our study regarding BMI.

Operative times in RCS were longer compared to LCS in our study. Prolonged operative times in RCS can be attributed to several factors such as docking time, learning curve and more technically demanding procedures like intracorporeal suturing. It has previously been reported that a surgeon’s learning curve influences surgery time. After 21 cases, the operative times in RCS and LCS in right-sided hemicolectomy for cancer were equalized [37]. We found the operation time was still prolonged in the RCS group after the implementation of CME surgery.

Overall complication rates and surgical morbidity has been described previously with varying results following RCS [14, 15, 59, 60]. Several factors are influenced by this outcome, why it is difficult to provide a conclusive explanation. Study’s data are heterogeneous and includes benign and malignant colorectal surgery, the results may be difficult to distinguish from each other and even misleading. The technical development has led to improved surgical procedures and reduced the risk of complications in robot-assisted surgery. It has several advantages over laparoscopic surgery and include a stable camera platform, the operator's possibility to control the camera independently, and the endowrist possibility of instruments [61]. The ever-increasing flexibility of the instruments makes it possible to perform complex procedures which either are impossible to perform or time-consuming by conventional laparoscopy. The tactile sense in open colorectal surgery is difficult to reproduce in minimally invasive surgery. However, RCS provides haptic feedback giving the surgeon a more “real” experience of the surgical procedure while performing intracorporeal suturing, manipulating the tissue and during dissection work [62]. This feature may help minimize the risk of intraoperative complications and reduce overall morbidity.

Time to first oral diet was reduced in the RCS group. This observation's significance can be debated since the reduction was minimal (mean difference < 1 day) in relation to the high heterogeneity across studies. Previous studies have demonstrated a difference in length of stay, time to first flatus and oral intake in favor of RCS [13, 14, 55]. Despite divergent results, it may be assumed that RCS causes less tissue trauma due to better hemostasis technique and haptic feedback resulting in less manipulation of the organs.

The strengths of this study were the extensive literature search despite the inclusion of only one RCT. By supplementing the main results with subgroup-, sensitivity-, and meta-regression analyses we explored potential sources of outcomes with substantial heterogeneity. In a subgroup analysis of right-sided colectomies, we demonstrated no difference in the overall complication rate and time to regular diet between the two groups compared to the results from primary analysis, while length of stay was reduced by approximately one day in the RCS group. Due to a limited number of studies performing left-sided colectomies conducting a subgroup analysis was impossible. We conducted a sensitivity analysis with the exclusion of outlier studies with extreme effect sizes, as a consequence of substantial and considerable heterogeneity that originated in some of our primary and secondary outcomes. An additional significant difference was demonstrated regarding the intraoperative blood loss and amount of harvested lymph nodes in favor of RCS.

A supplemental meta-regression analysis was performed to verify any moderator effect on primary outcomes. Higher age was proved to reduce the risk of conversion in robot-assisted surgery, while the male sex had a greater advantage if operated by the laparoscopic method. Additionally, an intracorporeal anastomotic technique predicted a reduction in operative time in the RCS group probably as a direct consequence of a more accessible approach to intracorporeal suturing than the LCS group. A more recent year of publication impacted conversion rates and was reduced in the RCS group, while the operative time constantly favored laparoscopic surgery across all years included in this meta-analysis. By a summary analysis of literature available, we identified several clinically relevant factors favoring RCS compared to LCS. This new information can be utilized for a transformation process and replace laparoscopic surgery in the treatment of colon cancer. Although some of the outcomes had little clinical significance (e.g., reduction in length of stay and time to initiation of regular diet), this is believed to be indirectly correlated to the lower overall complication-, conversion-, and anastomotic leakage rate favoring RCS.

The substantial limitation of this study is the inclusion of studies with a non-randomized design resulting in a risk of selection bias. A patient selection in favor of RCS may occur during the implementation phase explained by the differences in some of the preoperative demographic and clinical outcomes between RCS and LCS. The studies included in our meta-analysis were of a different design regarding the type of surgery and anastomotic techniques. A plausible explanation of high heterogeneity deriving in some of our outcomes of interest can be explained by this diversity. Since most of our studies are of an observational design the evidence level is very low, and risk of bias assessment of serious concern. Our results may likewise have been over- or underestimated regarding following outcomes: length of stay, time to first flatus, time to regular diet, rate of intraoperative bleeding thereby causing information bias.

In conclusion, the present meta-analysis indicates that RCS for colon cancer is superior to LCS in several intra- and postoperative outcomes with regard to a very low level of evidence and serious risk of bias. Our result can thus only be applied to right-sided resections due to lack of studies comparing exclusively left-sided colon surgery, why this surgical method cannot be recommended by default for this group of patients. Due to the considerable divergence between anastomosis techniques in right-sided resections, there is a need for prospective randomized studies examining intra- and extracorporeal techniques in RCS. It is believed that postoperative complication rates and time of convalescence are improved by intracorporeal anastomosis technique in RCS.

## Supplementary Information

Below is the link to the electronic supplementary material.

Supplementary file1 (DOCX 9827 kb)

Supplementary file2 (DOCX 818 kb)

Supplementary file3 (DOCX 254 kb)

Supplementary file4 (DOCX 1973 kb)

Supplementary file5 (DOCX 14 kb)

Supplementary file6 (DOCX 14 kb)

Supplementary file7 (DOCX 14 kb)

Supplementary file8 (DOCX 14 kb)

Supplementary file9 (DOCX 596 kb)

## Data Availability

Data extracted and included in the systematic review and meta-analysis can be handed out on request from the corresponding author.
