# Supplementary figures and images for: Short-term outcomes in robot-assisted compared to laparoscopic colon cancer resections: a systematic review and meta-analysis
Source: Surg Endosc. 2021 Nov 1;36(1):32–46. doi: 10.1007/s00464-021-08782-7 (PMC8741661; doi:10.1007/s00464-021-08782-7)

**Supplemental digital content 2**

**Figure 1: Risk of bias assessment of included studies**

**
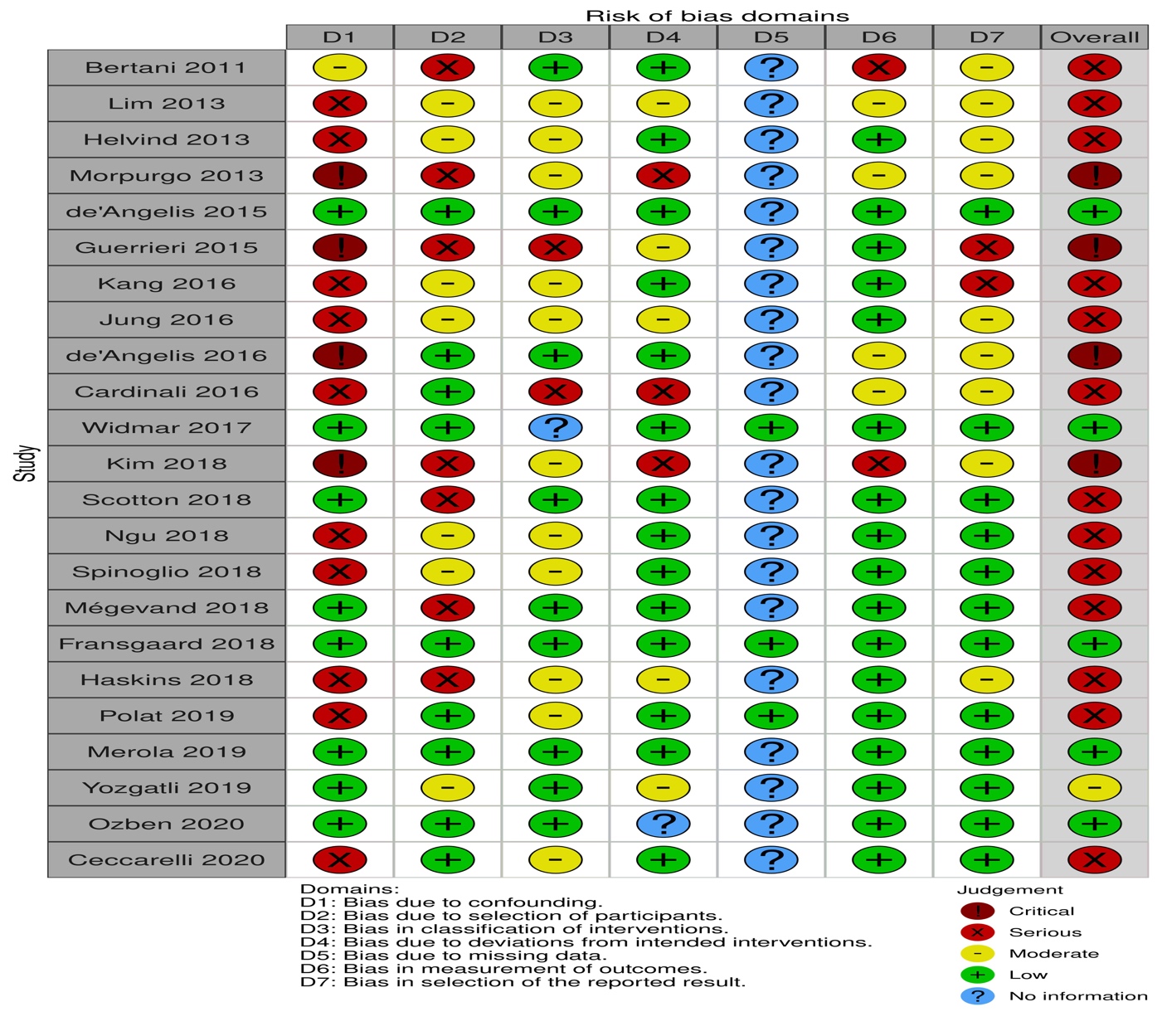
**


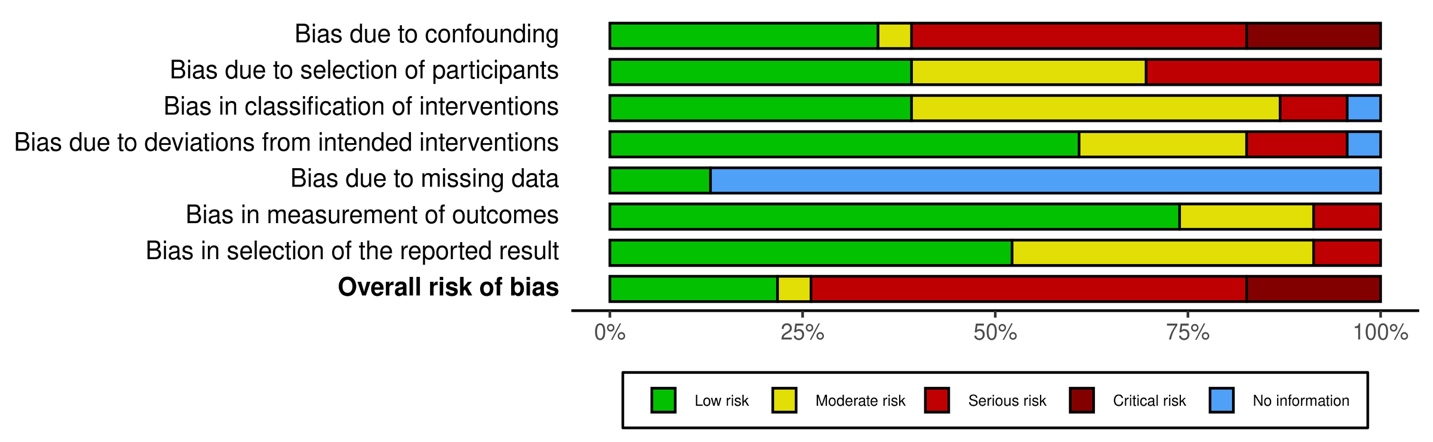

Supplement: Supplementary file 2 — Supplementary file2 (DOCX 818 kb) [file 464_2021_8782_MOESM2_ESM.docx]
