# Supplementary material for: Short-term outcomes in robot-assisted compared to laparoscopic colon cancer resections: a systematic review and meta-analysis
Source: Surg Endosc. 2021 Nov 1;36(1):32–46. doi: 10.1007/s00464-021-08782-7 (PMC8741661; doi:10.1007/s00464-021-08782-7)
Supplement: Supplementary file 3 — Supplementary file3 (DOCX 254 kb) [file 464_2021_8782_MOESM3_ESM.docx]

**Supplemental digital content 3**

**Figure 1: Forest plot of operative time**

R = right colectomy, L = left colectomy, M = right and left colectomy, CI = confidence interval, I^2^ = heterogeneity
