# Supplementary material for: Short-term outcomes in robot-assisted compared to laparoscopic colon cancer resections: a systematic review and meta-analysis
Source: Surg Endosc. 2021 Nov 1;36(1):32–46. doi: 10.1007/s00464-021-08782-7 (PMC8741661; doi:10.1007/s00464-021-08782-7)
Supplement: Supplementary file 4 — Supplementary file4 (DOCX 1973 kb) [file 464_2021_8782_MOESM4_ESM.docx]

**Supplemental digital content 4**

**Figure 1: Forest plot of overall complication rate**

R = right colectomy, L = left colectomy, M = right and left colectomy, CI = confidence interval, I^2^ = heterogeneity

**Figure 2: Forest plot of time to regular diet**

R = right colectomy, L = left colectomy, M = right and left colectomy, CI = confidence interval, I^2^ = heterogeneity

**Figure 3: Forest plot of time to first flatus**

R = right colectomy, L = left colectomy, M = right and left colectomy, CI = confidence interval, I^2^ = heterogeneity

**Figure 4: Forest plot of abdominal abscess**

****R = right colectomy, L = left colectomy, M = right and left colectomy, CI = confidence interval, I^2^ = heterogeneity

**Figure 5: Forest plot of Clavien Dindo grade of complications I-III**

R = right colectomy, L = left colectomy, M = right and left colectomy, CI = confidence interval, I^2^ = heterogeneity

**Figure 6: Forest plot of Clavien Dindo grade of complications IV-V**

R = right colectomy, L = left colectomy, M = right and left colectomy, CI = confidence interval, I^2^ = heterogeneity

**Figure 7: Forest plot of medical complication rate**

R = right colectomy, L = left colectomy, M = right and left colectomy, CI = confidence interval, I^2^ = heterogeneity

**Figure 8: Forest plot of postoperative bleeding rate**

R = right colectomy, L = left colectomy, M = right and left colectomy, CI = confidence interval, I^2^ = heterogeneity

**Figure 9: Forest plot of postoperative ileus**

R = right colectomy, L = left colectomy, M = right and left colectomy, CI = confidence interval, I^2^ = heterogeneity

**Figure 10: Forest plot of wound abscess**

R = right colectomy, L = left colectomy, M = right and left colectomy, CI = confidence interval, I^2^ = heterogeneity

**Figure 11: Forest plot of harvested lymph nodes**

R = right colectomy, L = left colectomy, M = right and left colectomy, CI = confidence interval, I^2^ = heterogeneity

**Figure 12: Forest plot of intraoperative blood loss**

R = right colectomy, L = left colectomy, M = right and left colectomy, CI = confidence interval, I^2^ = heterogeneity

**Figure 13: Forest plot of mortality rate**

R = right colectomy, L = left colectomy, M = right and left colectomy, CI = confidence interval, I^2^ = heterogeneity
