# Supplementary material for: Short-term outcomes in robot-assisted compared to laparoscopic colon cancer resections: a systematic review and meta-analysis
Source: Surg Endosc. 2021 Nov 1;36(1):32–46. doi: 10.1007/s00464-021-08782-7 (PMC8741661; doi:10.1007/s00464-021-08782-7)
Supplement: Supplementary file 5 — Supplementary file5 (DOCX 14 kb) [file 464_2021_8782_MOESM5_ESM.docx]

**Supplemental digital content 5**

**Table 1: Primary and secondary outcomes in a subgroup analysis of right-sided colectomies.**

| **Outcomes measurement** | **OR/MD** | **95% CI** | **I^2^** | **P-value** |
| --- | --- | --- | --- | --- |
|  |  |  |  |  |
| Medical complication rate | 1.00 | 0.69,1.46 | 0.25% | 0.98 |
| Clavien Dindo grade I-III | 1.21 | 0.85,1.71 | 0% | 0.28 |
| Clavien Dindo grade IV-V | 0.73 | 0.38,1.37 | 0% | 0.33 |
| Conversion rate | 0.18 | 0.10,0.30 | 12.72% | **0.00** |
| 30 days mortality | 1.17 | 0.43,3.16 | 0% | 0.76 |
| Anastomotic leakage | 0.41 | 0.19,0.88 | 0% | **0.02** |
| Abdominal abscess | 0.73 | 0.37,1.45 | 0% | 0.37 |
| Wound abscess | 0.87 | 0.57,1.31 | 0% | 0.51 |
| Postoperative bleeding | 0.97 | 0.60,1.58 | 15.18% | 0.91 |
| Postoperative ileus | 0.88 | 0.58,1.33 | 0% | 0.54 |
| Overall complication rate | 0.85 | 0.69,1.04 | 25.26% | 0.11 |
| Intraoperative blood loss | 11.74 | -19.40,42.87 | 78.40% | 0.46 |
| Harvested lymph nodes | 0.64 | -0.98,2.26 | 65.03% | 0.44 |
| Operative time | 57.45 | 38.47,76.42 | 94.46% | **0.00** |
| Time to regular diet | -0.29 | -0.62,0.04 | 57.15% | 0.09 |
| Length of stay | -0.91 | -1.60,-0.22 | 73.74% | **0.01** |
| Time to first flatus | -0.08 | -0.40,0.24 | 83.39% | 0.63 |

**OR = odds ratio, MD = mean difference. The reference value is the RCS group. Values (OR/MD) < 1 are in favor for the RCS group and > 1 for the LCS group.**
