# Supplementary material for: Short-term outcomes in robot-assisted compared to laparoscopic colon cancer resections: a systematic review and meta-analysis
Source: Surg Endosc. 2021 Nov 1;36(1):32–46. doi: 10.1007/s00464-021-08782-7 (PMC8741661; doi:10.1007/s00464-021-08782-7)
Supplement: Supplementary file 6 — Supplementary file6 (DOCX 14 kb) [file 464_2021_8782_MOESM6_ESM.docx]

**Supplemental digital content 6**

**Table 1: Sensitivity analysis on primary and secondary outcomes with substantial and considerable heterogeneity (I^2^ > 50) from the primary analysis.**

| **Outcomes measurement** | **OR/MD** | **95% CI** | **I^2^** | **P-value** |
| --- | --- | --- | --- | --- |
|  |  |  |  |  |
| Intraoperative blood loss | -17.14 | -24.41,-9.87 | 19.10% | **0.00** |
| Harvested lymph nodes | -0.96 | -1.79,-0.14 | 0% | **0.02** |
| Operative time | 41.99 | 35.01,48.96 | 0% | **0.00** |
| Time to regular diet | -0.26 | -0.52,0.01 | 0% | 0.06 |
| Length of stay | -0.45 | -0.73,-0.17 | 0% | **0.00** |
| Time to first flatus | -0.12 | -0.29,0.05 | 0% | 0.15 |

**OR = odds ratio, MD = mean difference. The reference value is the RCS group. Values (OR/MD) < 1 are in favor for the RCS group and > 1 for the LCS group.**
