# Supplementary material for: Short-term outcomes in robot-assisted compared to laparoscopic colon cancer resections: a systematic review and meta-analysis
Source: Surg Endosc. 2021 Nov 1;36(1):32–46. doi: 10.1007/s00464-021-08782-7 (PMC8741661; doi:10.1007/s00464-021-08782-7)
Supplement: Supplementary file 7 — Supplementary file7 (DOCX 14 kb) [file 464_2021_8782_MOESM7_ESM.docx]

**Table 1: Sensitivity analysis of studies with low risk of bias**

| **Outcomes measurement** | **OR/MD** | **95% CI** | **I^2^** | **P-value** |
| --- | --- | --- | --- | --- |
|  |  |  |  |  |
| Medical complication rate | 0.98 | 0.36,2.64 | 0% | 0.97 |
| Clavien Dindo grade I-III | 1.47 | 0.82,2.63 | 0% | 0.20 |
| Clavien Dindo grade IV-V | 0.76 | 0.31,1.86 | 0% | 0.55 |
| Conversion rate | 0.31 | 0.22,0.43 | 50.17% | **0.00** |
| 30 days mortality | 0.87 | 0.14,5.26 | 0% | 0.88 |
| Anastomotic leakage | 0.72 | 0.15,3.68 | 0% | 0.71 |
| Abdominal abscess | 0.74 | 0.23,2.33 | 0% | 0.60 |
| Wound abscess | 0.82 | 0.34,1.93 | 0% | 0.64 |
| Postoperative bleeding | 0.69 | 0.03,17.29 | 0% | 0.82 |
| Postoperative ileus | 0.58 | 0.21,1.61 | 0% | 0.30 |
| Overall complication rate | 0.89 | 0.71,1.11 | 0% | 0.30 |
| Intraoperative blood loss | 16.83 | -40.21,73.88 | 86.96% | 0.56 |
| Harvested lymph nodes | 1.44 | -1.69,4.57 | 76.01% | 0.37 |
| Operative time | 71.00 | 16.81,125.19 | 98.56% | **0.01** |
| Time to regular diet | NR | NR | NR | NR |
| Length of stay | NR | NR | NR | NR |
| Time to first flatus | NR | NR | NR | NR |

**OR = odds ratio, MD = mean difference, NR = not reported. The reference value is the RCS group. Values (OR/MD) < 1 are in favor for the RCS group and > 1 for the LCS group.**
