# Supplementary material for: Short-term outcomes in robot-assisted compared to laparoscopic colon cancer resections: a systematic review and meta-analysis
Source: Surg Endosc. 2021 Nov 1;36(1):32–46. doi: 10.1007/s00464-021-08782-7 (PMC8741661; doi:10.1007/s00464-021-08782-7)
Supplement: Supplementary file 8 — Supplementary file8 (DOCX 14 kb) [file 464_2021_8782_MOESM8_ESM.docx]

**Supplemental digital content 8**

**Table 1: Meta-regression analysis of potential demographic and clinical moderator effects on primary outcomes with RCS as reference compared to LCS group.**

| **Outcome** | **Moderator variable** | **Regression Coefficeint** | **CI (lower limit)** | **CI (upper limit)** | **p-value** |
| --- | --- | --- | --- | --- | --- |
|  |  |  |  |  |  |
| **Anastomotic leakage** | Previous surgery | -1.782 | -6.218 | 2.654 | 0.431 |
|  | BMI | -0.269 | -1.007 | 0.469 | 0.475 |
|  | Anastomosis technique | -0.113 | -2.136 | 1.910 | 0.913 |
|  | Age | -0.187 | -0.563 | 0.189 | 0.331 |
|  | Tumor | -0.026 | -0.789 | 0.737 | 0.947 |
|  | Male | 0.092 | -0.191 | 0.376 | 0.524 |
|  | Publication year | -0.162 | -0.366 | 0.042 | 0.120 |
|  |  |  |  |  |  |
| **Conversion rate** | Previous surgery | 0.324 | -4.930 | 5.579 | 0.904 |
|  | BMI | 0.218 | -0.680 | 1.116 | 0.635 |
|  | Anastomosis technique | 0.416 | -2.415 | 3.247 | 0.773 |
|  | Age | -0.407 | -0.727 | -0.087 | **0.013** |
|  | Tumor | -0.087 | -0.977 | 0.803 | 0.848 |
|  | Male | 0.431 | 0.045 | 0.818 | **0.029** |
|  | Publication year | -0.225 | -0.452 | 0.002 | **0.052** |
|  |  |  |  |  |  |
| **Operative time** | Previous surgery | -0.897 | -4.539 | 2.745 | 0.629 |
|  | BMI | 0.068 | -0.445 | 0.581 | 0.795 |
|  | Anastomosis technique | -0.860 | -1.576 | -0.144 | **0.019** |
|  | Age | -0.017 | -0.242 | 0.208 | 0.881 |
|  | Tumor | 0.205 | -0.342 | 0.752 | 0.463 |
|  | Male | -0.188 | -0.565 | 0.189 | 0.327 |
|  | Publication year | 0.154 | -0.003 | 0.312 | **0.054** |
|  |  |  |  |  |  |
| **Length of stay** | Previous surgery | 0.027 | -2.962 | 3.016 | 0.986 |
|  | BMI | -0.178 | -0.623 | 0.263 | 0.426 |
|  | Anastomosis technique | -0.181 | -0.491 | 0.128 | 0.251 |
|  | Age | -0.094 | -0.316 | 0.128 | 0.407 |
|  | Tumor | 0.102 | -0.369 | 0.574 | 0.670 |
|  | Male | 0.059 | -0.269 | 0.389 | 0.724 |
|  | Publication year | -0.036 | -0.185 | 0.113 | 0.636 |
