# Supplementary material for: Short-term outcomes in robot-assisted compared to laparoscopic colon cancer resections: a systematic review and meta-analysis
Source: Surg Endosc. 2021 Nov 1;36(1):32–46. doi: 10.1007/s00464-021-08782-7 (PMC8741661; doi:10.1007/s00464-021-08782-7)
Supplement: Supplementary file 9 — Supplementary file9 (DOCX 596 kb) [file 464_2021_8782_MOESM9_ESM.docx]

**Supplemental digital content 9**

**Table 1: Eggers’ linear regression test for publication bias**

| **Outcome measurement** | **Regression coefficient** | **95% CI** | **P-value** |
| --- | --- | --- | --- |
|  |  |  |  |
| Abdominal abscess | 0.34 | -1.23,1.91 | 0.67 |
| Anastomotic leakage | 0.33 | -1.05,1.73 | 0.64 |
| Clavien Dindo grade I-III | 0.62 | -1.56,2.79 | 0.58 |
| Clavien Dindo grade IV-V | 0.25 | -1.95,2.46 | 0.82 |
| Overall complication rate | 0.35 | -0.72,1.43 | 0.52 |
| Conversion rate | 0.55 | -0.62,1.73 | 0.36 |
| Harvested lymph nodes | 1.70 | 0.29,3.12 | **0.02** |
| Intraoperative blood loss | 2.66 | 1.39, 3.94 | **< 0.001** |
| Length of stay | 1.48 | -0.43,3.40 | 0.13 |
| Medical complication rate | -0.34 | -1.58, 0.91 | 0.59 |
| Mortality rate (30 days) | 0.38 | -1.74,2.49 | 0.73 |
| Operative time | 2.33 | -2.08,6.74 | 0.30 |
| Postoperative bleeding | -0.31 | -1.52, 0.89 | 0.61 |
| Postoperative ileus | -0.34 | -1.41, 0.74 | 0.54 |
| Time to first flatus | 0.37 | -1.95,2.68 | 0.76 |
| Time to regular diet | -0.55 | -2.48, 1.37 | 0.57 |
| Wound abscess | 0.90 | -0.26, 2.05 | 0.13 |

**CI = confidence interval**

**Figure 1: Funnel plot of medical complication rate**

**Figure 2: Funnel plot of Clavien Dindo grade I-III**

**Figure 3: Funnel plot of Clavien Dindo grade IV-V**

**Figure 4: Funnel plot of conversion rate**

**Figure 5: Funnel plot of 30 days mortality**

**Figure 6: Funnel plot of anastomotic leakage**

**Figure 7: Funnel plot of abdominal abscess**

**Figure 8: Funnel plot of wound abscess**

**Figure 9: Funnel plot of postoperative bleeding**

**Figure 10: Funnel plot of postoperative rate of ileus**

**Figure 11: Funnel plot of overall complication rate**

**Figure 12: Funnel plot of operative time**

**Figure 13: Funnel plot of intraoperative blood loss**

**Figure 14: Funnel plot of harvested lymph nodes**

**Figure 15: Funnel plot of time to regular diet**

**Figure 16: Funnel plot of length of stay**

**Figure 17: Funnel plot of time to first flatus**
